# DyP-Type Peroxidases: Recent Advances and Perspectives

**DOI:** 10.3390/ijms22115556

**Published:** 2021-05-24

**Authors:** Yasushi Sugano, Toru Yoshida

**Affiliations:** Department of Chemical and Biological Sciences, Faculty of Science, Japan Women’s University, Tokyo 112-8681, Japan; yoshidat@fc.jwu.ac.jp

**Keywords:** DyP, DyP-type peroxidase, structure-based sequence alignments, antifungal anthraquinone compounds, lignin degradation, iron uptake, life cycle, hydrolase, oxidase, encapsulin, nano compartment, cargo protein

## Abstract

In this review, we chart the major milestones in the research progress on the DyP-type peroxidase family over the past decade. Though mainly distributed among bacteria and fungi, this family actually exhibits more widespread diversity. Advanced tertiary structural analyses have revealed common and different features among members of this family. Notably, the catalytic cycle for the peroxidase activity of DyP-type peroxidases appears to be different from that of other ubiquitous heme peroxidases. DyP-type peroxidases have also been reported to possess activities in addition to peroxidase function, including hydrolase or oxidase activity. They also show various cellular distributions, functioning not only inside cells but also outside of cells. Some are also cargo proteins of encapsulin. Unique, noteworthy functions include a key role in life-cycle switching in *Streptomyces* and the operation of an iron transport system in *Staphylococcus aureus, Bacillus subtilis* and *Escherichia coli*. We also present several probable physiological roles of DyP-type peroxidases that reflect the widespread distribution and function of these enzymes. Lignin degradation is the most common function attributed to DyP-type peroxidases, but their activity is not high compared with that of standard lignin-degrading enzymes. From an environmental standpoint, degradation of natural antifungal anthraquinone compounds is a specific focus of DyP-type peroxidase research. Considered in its totality, the DyP-type peroxidase family offers a rich source of diverse and attractive materials for research scientists.

## 1. Introduction

**Figure 1:**
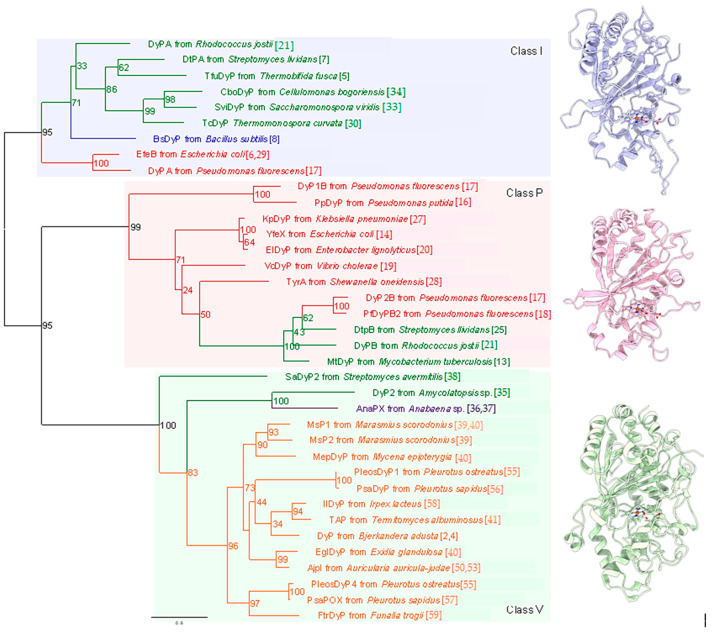
DyP phylogenetic tree. The tree was constructed with the maximum-likelihood method using RAxML-NG [61] with multiple sequence alignments of DyP amino acid sequences generated by the MAFFT [62] program. Percentages of bootstrap values obtained from 1000 bootstrap replicates are shown at the nodes. The best-fit model of evolution of the alignment was determined using ModelTest-NG [63]. Branches and labels of different phyla are shown in different colors: green, actinobacteria; blue, firmicutes; red, proteobacteria; purple, cyanobacteria; orange, basidiomycota. Reference numbers for each DyP are shown in parentheses. Structures of representative DyPs of the three classes are shown on the right: Class I, DtPA (PDB ID: 6gzw); Class P, DtPB (PDB ID: 6yrj); Class V: DyP (PDB ID: 3afv).

General peroxidase research has seemed to wane in recent years, at least in part because peroxidases are ubiquitous and their generally accepted physiological role in plants—removing hydrogen peroxide (H_2_O_2_) generated inside of cells and constructing a part of the cell wall such as lignin—has been long and thoroughly studied. In contrast, the research history of DyP-type peroxidases is in its early stages, with the first identification of the enzyme occurring only two decades ago. The first DyP-type peroxidase, dye decolorizing peroxidase (DyP) from *Geotrichum candidum*, was reported in 1999 [1,2]. The origin of this enzyme was later revised to *Bjerkandera adusta* [3]. In 2007, DyP-type peroxidases were recognized as being distinguishable from other known peroxidase and were defined as a novel peroxidase family—the DyP-type peroxidase family [4]. To date, DyP-type peroxidases have been found in a variety of organisms (Figure 1). These enzymes also exhibit a varied distribution, functioning not only inside but also outside of cells. In prokaryotes, in particular, the cellular distribution ranges widely from intracellular to extracellular, including periplasmic space. Some secreted DyP-type peroxidases have a Tat (twin arginine translocation) signal, suggesting that they are Tat substrates [5,6,7,8,9,10]. Notably, all secreted DyP-type peroxidases with Tat signal belong to class I, as described in a later section. Moreover, EfeB and YwbN, which are representative DyP-type peroxidases as Tat substrates, reside in the periplasmic space and outside of the cell, respectively. They are homologs of each other and are inferred to play similar physiological roles [8,11]. Among eukaryotes, basidiomycetes have been a source of interesting findings on DyP-type peroxidases. Surprisingly, the DyP-type peroxidases in these organisms are all extracellular, similar to some other peroxidases from basidiomycete. If the main role of DyP-type peroxidases is to scavenge the H_2_O_2_ generated inside of the cell or construct a part of cell tissues, it would be unnecessary to secrete the protein into the extracellular milieu. Therefore, secreted DyP-type peroxidases likely serve a function independent of removal of intracellular H_2_O_2_ or construct the cell tissues. Interestingly, it appears that some DyP-type peroxidases exist as a cargo protein in encapsulin [12,13]. In particular, there is speculation that MtDyP (class P) provides defense against attacks of host cells. Several DyP-type peroxidases are summarized in outline form in Table 1.

In any case, research performed over the past decade has clarified the unique characteristics of DyP-type peroxidases, converging on the view that they do not exist solely to remove H_2_O_2_ but instead have more important specific roles.

## 2. Importance of Tertiary Structure and Catalytic Mechanism

### 2.1. Characteristics Based on Tertiary Structure

In early-stage research, DyP-type peroxidases were categorized into classes A, B, C and D according to primary structural homologies (RedOxiBase). Although this orthodox classification is useful, it has limited value for studying catalytic mechanisms and active sites because the primary structure provides only amino acid sequence information. In contrast, tertiary structures define tangible entities that allow for detailed assessments of the relationship between structure and function among members of the DyP-type peroxidase family. In 2015, a new classification scheme was proposed for this family based on structure-based sequence alignments obtained using the multiple three-dimensional alignment tool, MATRAS [42]. Such tertiary structural analyses are apparently effective for further analysis because they revealed genuine structural homologies among different DyP-type peroxidases. Interestingly, this new scheme clearly reclassified the family into three new classes, combining classes C and D into a new class V (adVanced) and renaming classes A and B as classes I (Intermediate) and P (Primitive), respectively [42]. Throughout this review, we apply this new class I, P and V classification scheme. Typical structures of each of the three classes are shown on the right side of Figure 1.

The basic skeletal structure is common among the three classes and consists of a dimeric α + β barrel structure [42]. The structures of classes I and V are constructed based on class P, with some additional regions. Therefore, class P members have the smallest molecular size among the three classes, with members of a class V having the largest size. Notably, all important amino acid residues that define DyP-type peroxidases are included in the skeletal structure (i.e., class P), suggesting that basic functions are determined by this skeletal structure, with extra regions mediating additional functions. For instance, catalytic efficiencies (*k*_cat_/*K*_m_) toward anthraquinone compounds are different among the three classes. As shown in Table 1, the catalytic efficiencies of most class V members (10^4^ to 10^7^) are the largest among the three classes. By comparison, catalytic efficiencies range from 10^3^ to 10^6^ for class I and from 10^2^ to 10^5^ for class P.

### 2.2. Catalytic Mechanism

**Figure 2:**
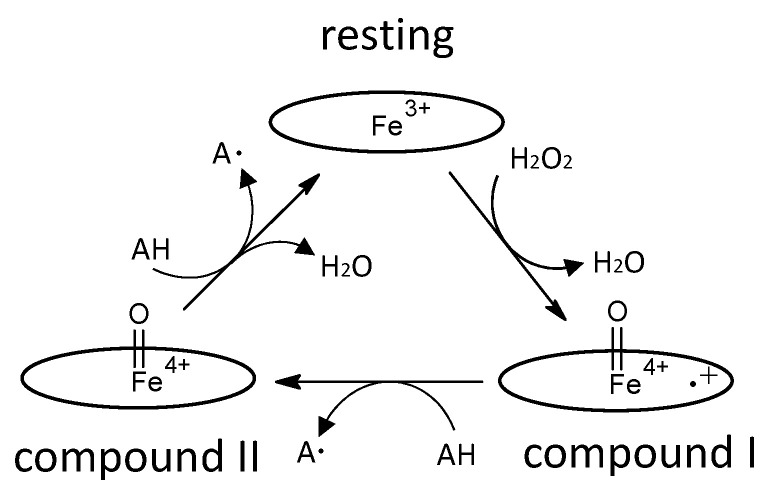
Catalytic cycle of a typical heme peroxidase. Oval denotes the heme plane in the enzyme. AH is a substrate.

**Figure 3:**
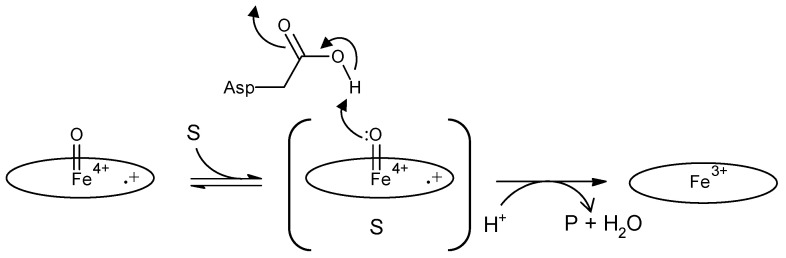
Schematic diagram of two-electron oxidation of substrates by a DyP-type peroxidase, proposed in [20]. Left, compound I; center, ES complex; right, resting state. Oval denotes the heme plane in the enzyme. Asp is the catalytic residue. S and P denote substrate and product, respectively. In a general peroxidase, compound I changes to compound II with one electron reduction, as shown in Figure 2, but in this scheme, compound I of DyP-type peroxidases changes to the resting state directly through two-electron reduction.

The enzyme commission number for DyP is EC 1.11.1.19, which corresponds to a peroxidase group. Actually, in most cases, DyP-type peroxidases show typical peroxidase activity, such as that shown by horseradish peroxidase (HRP). Therefore, the catalytic cycle appears to be nearly the same as that of ubiquitous peroxidases [64]. As shown in Figure 2, general heme peroxidases cycle through resting, compound I and compound II states. The compound I formation process of DyP-type peroxidase is essentially inconsistent with that of classical heme peroxidases such as lignin peroxidase [4]. In the case of DyP-type peroxidase, resting and compound I states have been confirmed, but compound II is controversial. DyPA (class I) from *Rhodococcus jostii* shows a spectrum corresponding to compound II at pH 7.5 [21]. TcDyP (class I) from *Thermomonospora curvata* shows compound II at pH 7.8 with both H_2_O_2_ and hydroquinone, but not with H_2_O_2_ only [30]. Furthermore, compound II was not observed at pH 3, which is the optimum pH for dye decolorizing [30]. In support of this, Shrestha et al. reported that compound II was not observed at low pH in ElDyP (class P) from *Enterobacter lignolyticus*, suggesting that the catalytic cycle does not follow a typical two-step process [20]. The authors of this latter study suggested that the enzyme adopts a two-electron reduction process for compound I, as shown in Figure 3. Moreover, no exact compound II has yet been reported at low pH for DyP-type peroxidases belonging to classes P or V. Therefore, further study at low pH—the active pH for dye decolorization—is necessary regardless of whether the two-step catalytic cycle with sequential one-electron reduction of the enzyme occurs or not. On the other hand, excess H_2_O_2_ deactivates DyP-type peroxidsaes, a property also characteristic of ubiquitous peroxidases.

### 2.3. Active or Binding Sites

DyP-type peroxidases show a broad range of substrate specificities, and several active sites have been proposed [31,43,49,65]. It is likely that the binding site(s) of most substrates are different from the H_2_O_2_ binding site. We will first focus on the H_2_O_2_ binding site, which is located at the distal site of the heme. As described previously, aspartic acid and arginine are absolutely conserved in the H_2_O_2_-binding site of most DyP-type peroxidases, whereas general peroxidases have histidine and arginine at this site [44]. In particular, aspartic acid (D) in the GXXDG motif, which is a well-known conserved region among DyP-type peroxidases, replaces the histidine in general peroxidases, accounting for the lower optimum pH of DyP-type peroxidases compared with general peroxidases [4,44]. A D171N point mutation in DyP (class V) from *B. adusta* results in the disappearance of compound I formation. This causes a drastic decrease in peroxidase activity [4], highlighting the critical importance of aspartic acid in GXXDG. In contrast, Singh et al. reported that the arginine in DyPB (class P) from *R. jostii* is essential for peroxidase activity [22]. It has similarly been found that the arginine in DtpB (class P) from *Streptomyces lividans* is also a key residue for peroxidase activity [25]. In this latter study, a serial femtosecond X-ray crystallography approach was used to determine how the distal heme site in DyP-type peroxidase can be tuned to choose either aspartic acid or arginine. In the case of YfeX (class P), the activity toward guaiacol and catechol is retained if the arginine is replaced with other amino acids, suggesting that this arginine may play a limited role [14]. Additionally, it has been reported that the distal aspartic acid of ElDyP (class P) is catalytically more important than the distal arginine and plays a key role in determining the acidic pH optimum of DyPs [20]. Similar effect depending on pH has been reported in a resurrect class V DyP [60].

As noted above, it is clear that DyP forms compound I, indicating two-electron oxidization using hydrogen peroxide. Actually, compound I of DtpB has been directly determined for a peroxidase carrying a porphyrin π cation radical [25]. Although compound I oxidizes various substrates, no reports provide direct evidence to show how and where substrates bind to it. This is one of the least clarified and essential characteristics of DyP-type peroxidases. The active sites of substrates other than H_2_O_2_ are probably located apart from the distal heme region, which would otherwise limit access to bulky molecules, such as synthetic dyes. A likely hypothesis is that this site is located at the molecular surface of the enzyme, and that an electron is transferred from the distal area of the heme to the substrate via a long-range electron-transfer route [31,49,66]. A similar speculation has been raised for versatile peroxidases [67]. Several studies have shown that aromatic residues such as tryptophan and tyrosine accumulate and retain radicals, thereby serving as a point of substrate oxidation [19,31,49,66]. This hypothesis seems reasonable, although unfortunately there are no reports of a genuine enzyme–substrate (ES) complex between bulky substrates and aromatic residues in DyP-type peroxidases. In contrast, DMP (2,6-dimethoxyphenol), which is a general substrate for peroxidases, has been reported to associate with asparagine 313 at the molecular surface of DyP from *B. adusta* (class V), and a hydrogen-bonding network has been reported from there to the propionate of heme [43]. This network is similar to that of ascorbate peroxidase [68]. To date, this is the sole report of an ES complex for a DyP-type peroxidase. Although a Mn^2+^ binding pocket was observed in an artificial N246A mutant of DypB [23], there was no report of an ES complex in the case of native DypB. Despite these advances, the location of the binding site for bulky substrates such as synthetic dyes, the representative substrates of DyP, has remained elusive.

## 3. Functions Besides Peroxidase

### 3.1. Hydrolase

**Figure 4:**
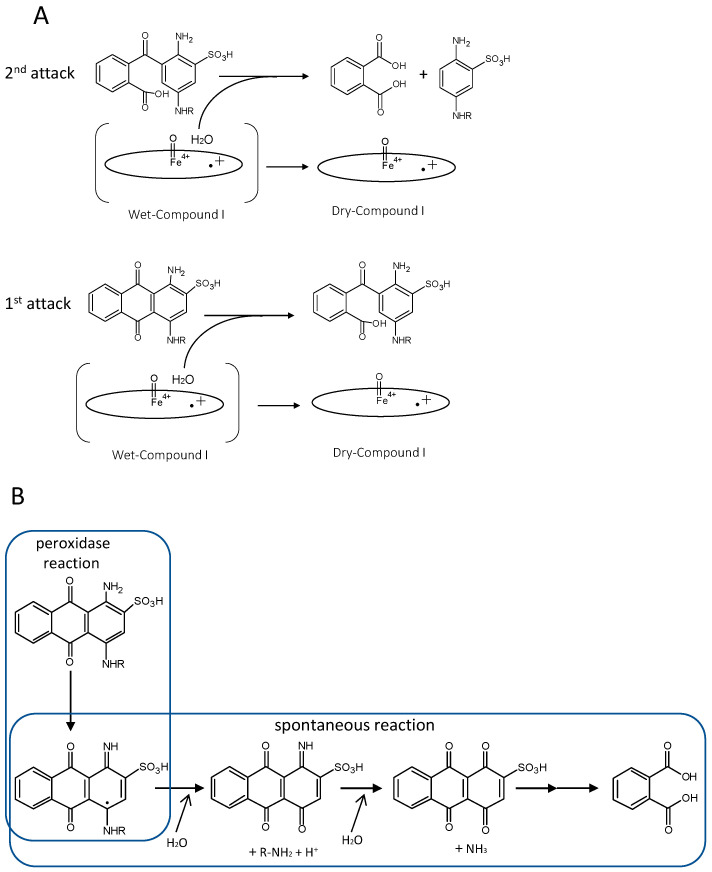
Comparison of proposed anthraquinone degradation mechanisms. A, H_2_O from Wet compound I attacks the anthraquinone frame, causing enzymatic hydrolysis [44]. Oval denotes the heme plane in the enzyme. B, Spontaneous hydrolysis generates phthalic acid, but predicted intermediates have not been detected [66].

Some DyP-type peroxidases have been reported to have other functions besides peroxidase activity. One of the reaction products confirmed to arise from catalysis of Reactive blue 5—a representative anthraquinone dye—by DyP from *Bjerkandera adusta* is phthalic acid [44,45]. This indicates that the anthraquinone frame is degraded by hydrolysis. If this is the case, it is reasonable that DyP acts as a hydrolase because this hydrolysis would never proceed in the absence of H_2_O_2_, suggesting the necessity of forming H_2_O from H_2_O_2_. The importance of H_2_O produced from H_2_O_2_ is also reported for classical heme peroxidases and catalases. Jones reported that wet (H_2_O-containing) and dry (non-H_2_O-containing) forms of compound I apparently play independent roles. He proposed a redox pathway switching mechanism, such that the states for the two electron-equivalent reduction of compound I are accessible in the dry form, but in the wet form only one-electron-equivalent processes are possible, unless the release of water can be stimulated [69]. The importance of a water molecule in the heme distal area was also reported for DtpB from *S. lividans* (class I) [25]. These reports suggested that H_2_O from H_2_O_2_ might play a specific role in the catalytic mechanism. Together, these observations suggest that DyP from *B. adusta* is a bifunctional enzyme that exerts hydrolase activity using the H_2_O released by the peroxidase function. In contrast, Linde et al. have reported that spontaneous hydrolysis would occur if an anthracenetetrone was formed as an intermediate from an anthraquinone, given that the ubiquitous heme peroxidase HRP also decolorizes Reactive blue 5 [66]. Actually, HRP decolorizes Reactive blue 5, but there is no evidence that it degrades the anthraquinone frame because phthalic acid has thus far not been detected. It is probably the case that decolorization with HRP depends on degradation, not of the anthraquinone frame, but of an auxochrome group. Moreover, there are no reports that anthracenetetrone is generated as an intermediate for a DyP-type peroxidase. These two ideas are summarized in Figure 4.

### 3.2. Deferrochelatase or Iron Uptake

**Figure 5:**
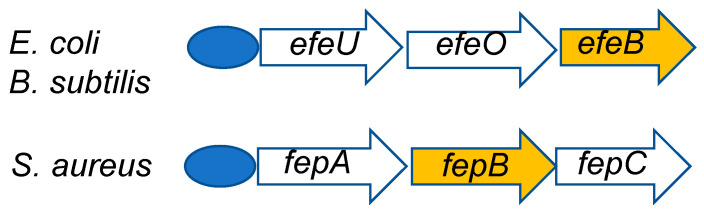
Schematic depiction of *efeUOB* and *fepABC* operons. Both operons are regulated by the Fur repressor (blue oval). *efeB* and *fepB* are orthologs of each other and encode DyP-type peroxidases.

In 2009, Létoffé et al. reported that EfeB (class I), a DyP-type peroxidase from *Escherichia coli*, functions as a deferrochelatase [70]. They observed that EfeB captures only iron without degradation of heme, which retains an intact tetrapyrrole skeleton. This phenomenon is quite different from that of heme oxygenase, which degrades the heme skeleton and then “picks out” the iron. This study created a profound impression on many researchers of DyP-type peroxidases. However, the corresponding function of FepB (class I), an EfeB homolog from *Staphylococcus aureus*, is obscure [11]. Moreover, YwbN (class I) from *Bacillus subtilis*, also a homolog of EfeB, shows no deferrochelatase activity, but does exhibit peroxidase activity [8]. Overall, whether DyP-type peroxidases have deferrochelatase activity remains a matter of controversy. Viewed from another standpoint, EfeB is a part of the EfeUOB operon, which encodes components of an iron transport system [29]. Interestingly, a similar operon has been found in both *B. subtilis* and *S. aureus* [8,11], and it has been proposed that both EfeB homologs are typical peroxidases that result in oxidation of Fe^2+^. Moreover, the *efeB* gene of *B. subtilis* is under additional control of several σ factors that are associated with the cell envelope stress response, suggesting a key role in the life-cycle of the organism [8]. Collectively, these studies suggest that the relationship between iron uptake and the role of DyP-type peroxidases must be critical to bacterial viability because iron uptake is essential for life. Further study of this relationship can be expected. A schematic diagram of the EfeUOB operon from the above three organisms is shown in Figure 5.

### 3.3. Oxidase

Peroxidase reactions absolutely depend on peroxides such as H_2_O_2_. Surprisingly, MscDyP from *Marasmius scorodonius*, a typical class V DyP, appears to oxidize β-carotene without H_2_O_2_ [39]. If this proves to be the case, it means that MscDyP not only shows peroxidase activity but also possesses oxidase activity, suggesting a bifunctional enzyme. Additionally, catalases, which are similar to heme peroxidases, are also suggested to show phenol oxidase activity in the absence of H_2_O_2_ [71]. However, it has been reported that PsaPOX (class V) from *Pleurotus sapidus* oxidizes β-carotene in the presence of H_2_O_2_, suggesting peroxidase activity [57]. Strikingly, β-carotene was found to be transformed by recombinant PsaDyP in both the presence and absence of H_2_O_2_, although enzymatic activity was increased by the addition of H_2_O_2_ [56]. This suggests that rPsaDyP has an oxidase function in addition to peroxidase activity. PsaPOX was also shown to oxidize several alkenes, such as anethol to anisaldehyde, but the details of the reaction mechanism are still obscure [57].

## 4. Physiological Role of DyP-Type Peroxidases

One of the accepted physiological role of a plant peroxidase is to form lignin of cell wall consuming H_2_O_2_ generated inside of cells. In contrast, it is noteworthy that many DyP-type peroxidases function in the extracellular environment. This suggests that the main physiological role of these peroxidases is not removal of intracellular H_2_O_2_, leading to speculation about other cryptic roles. An additional noteworthy characteristic of DyP-type peroxidases is that they are distributed across a broad range of organisms, suggesting possible divergent physiological roles. Probable physiological roles are considered below.

### 4.1. Anthraquinone Degradation

The DyP (class V) from *Bjerkandera adusta* Dec 1 is a classic member of this family. This strain was isolated from soil in Japan and exhibits broad decolorizing effects on various synthetic dyes [72]. Notably, DyP showed stronger decolorizing actions against anthraquinone dyes than azo dyes, a unique characteristic compared with other decolorizing enzymes known to date, which have been reported to be effective toward azo dyes [73,74,75,76]. One of the most distinctive characteristics of DyP from *B. adusta* is its unique ability to degrade the anthraquinone skeleton. Reactive blue 5 is actually completely decolorized through concerted reactions with versatile peroxidases and DyP [46], making these enzymes suitable for treating wastewater containing synthetic dyes. However, such synthetic dyes are not true substrates because they are never generated in nature, suggesting that natural anthraquinone compounds must be true substrates. For example, plants, including trees, synthesize multiple anthraquinone compounds that serve antifungal functions. In this context, DyP from *B. adusta* degrades alizarin, which is a natural antifungal anthraquinone compound produced by plants [47]. *B. adusta* is a white rot fungus that parasitizes living trees, which in response generate phytoalexin to protect against infection. Therefore, one probable physiological role is to degrade antifungal anthraquinone compounds and accelerate tree parasitism. This is the only study of its kind to date, but future work on this issue is expected.

### 4.2. Lignin Degradation

A relationship between lignin degradation and some DyP-type peroxidases has been reported. In particular, there are many reports of this function for class V DyPs, as shown in Table 1. To date, lignin peroxidase (LiP), versatile peroxidase (VP), manganese peroxidase (MnP), and laccase (Lac) from basidiomycetes and ascomycetes have been well studied, as have lignin-degrading enzymes from basidiomycetes, such as white rot fungi [67,76,77,78]. In recent years, several DyP-type peroxidases have been found to degrade lignin or its model compounds [30,79,80,81]. Interestingly, lignin degradation by DyP-type peroxidases has been reported for bacteria as well as basidiomycetes, whereas general lignin-degrading enzymes are mainly isolated from basidiomycetes. DyPA from *Pseudomonas fluorescens* pf-5 [17], TfuDyP from *Thermobifida fusca* [5], and SviDyP from *Saccharomonospora viridis* DSM43017 [33] are class I, whereas DyP1B from *Pseudomonas fluorescens* pf-5 [17], DyP2B, and DyPB from *Rhodococcus jostii* are class P. DypB from *Rhodococcus jostii,* which is an actinomycete, has been reported to degrade lignin in the presence of Mn^2+^ and H_2_O_2_, but its activity is low compared with that of fungal lignin-degrading enzymes [24,35,66]. DypB might be a manganese peroxidase whose primary role is to oxidize Mn^2+^ to Mn^3+^ [24]. However, the *k*_cat_/*K*_m_ ratio of DypB for Mn^2+^ was reported to be 1/31,250th of that of a typical MnP, indicating that Mn^2+^ is unlikely an essential substrate for DypB [82]. In contrast, DypA (class I) from the same strain shows no lignin-degrading activity, suggesting a difference in physiological roles, despite the fact that both are DyP-type peroxidases [24]. More puzzlingly, TfuDyP (class I) from *T. fusca*, which is also an actinomycete, was shown to degrade kraft lignin and oxidize a β-aryl ether lignin model compound [32]. Specifically, this latter study demonstrated that *Streptomyces* sp. S6 grew in medium containing kraft lignin as a sole carbon source and showed apparent LiP activity. However, in the case of DyP, S6 showed low activity—at most 1% compared with LiP [81]. SviDyP from *Saccharomonospora viridis* showed optimum pH and temperatures of pH 7.0 and 70 °C, respectively. These characteristics seem to offer advantages compared with other DyP-type peroxidases for practical biobleaching of kraft pulp [33]. Furthermore, two lignin-degrading bacteria, *Ochrobacterium* sp. and *Paenibacillus* sp., which contain no class P DyP-type peroxidases that have been implicated in lignin degradation in other bacteria, alternatively possess a multi-copper oxidase gene, which shows oxidation activity for β-aryl ether and biphenyl lignin dimer model compounds [83]. Thus, the importance of DyP-type peroxidases in lignin degradation, at least in bacteria, is open to interpretation.

The situation is different in basidiomycetes, for which considerable research supports lignin degradation. White rot fungi, such as *Phanerochaete chrysosporium* and *Pleurotus ostreatus*, in particular have demonstrated prominent lignin degradation activity. Their main enzymes are LiP, MnP and VP. Notably, *P. chrysosporium* does not have a DyP-type peroxidase. Since DyP-type peroxidases were first identified, their potential for lignin degradation has been a focus of research attention [39,40,49,50,51,52,84]. Such studies have confirmed the widespread transcript-level expression of DyP-type peroxidases in almost all samples of fungi from forest floor habitats [85]. The class V DyP-type peroxidases, AjP I, AjP II, EglDyP and MepDyP, from basidiomycetes are secreted outside the cell and degrade non-phenolic lignin model compounds through their peroxidase activity [40,50]. However, the lignin-degrading activity of DyP-type peroxidases from basidiomycetes is at most 4% that of LiP from *P. chrysosporium* [40,66]. IlDyP (class V) from *Irpex lacteus* has been characterized and suggested to hydrolyze wheat straw [86]. Because wheat straw contains lignocellulose, IlDyP might function in lignin degradation. Consistent with this, IlDyP-1 and IlDyP-2 were reported to degrade the lignin model compound, DMP [87], although this study reported no data on degradation of lignin itself by IlDyP.

Taken together, these observations indicate that most lignin-degrading organisms that possess DyP also have general lignin-degrading enzymes, such as LiP, MnP, VP and multi-copper oxidase; thus, whether lignin-degrading activity is an essential function of DyP-type peroxidases remains obscure.

### 4.3. Life Cycle of an Actinomycete

DtpA (class I) from the actinomycete *S. lividans* appears to have a specific role in the life cycle of the organism. In *S. lividans*, copper is important for stimulating the developmental switch between vegetative mycelium and aerial hyphae. DtpA uses H_2_O_2_ to oxidize Cu^+^ to Cu^2+^, which causes maturation of GlxA; thus, DtPA is required for maturation of GlxA. GlxA, in turn, generates H_2_O_2_ from O_2_, thereby providing the H_2_O_2_ necessary for DtPA-mediated oxidation of Cu^+^ to Cu^2+^. GlxA is a key protein in the shift from vegetative mycelium to aerial hyphae [88]. Mature GlxA acts in concert with the cellulose synthase-like protein, CslA, to form an extracellular glycan specific for aerial hyphae. This role for DtpA in oxidizing metal ions would thus be similar to the function of EfeB, which oxidizes Fe^2+^ to Fe^3+^ [8]. Notable in this context, *S. lividans* also appears to have an *efeB* homologue, which is located in a gene cluster harboring an iron transporter. *Streptomyces avermitilis* also has an *efeB* homologue (SAV_5925), strongly suggesting its specific role.

### 4.4. Cargo Protein of Encapsulin

An additional unique characteristic of several DyPs is their function as a cargo protein for encapsulin, a bacterial nanocompartment protein. One of the best-studied of these DyPs is MtDyP (class P) from *Mycobacterium tuberculosis* [13,26], a bacterium that slips through defenses generated by the host immune response and induces tuberculosis. MtDyP, which is shielded from oxidative stress in the nanocompartment through association with encapsulin, appears to play an important role in defending against the immune assault by virtue of its peroxidase activity. DypB (class P) from *R. jostii* was shown to assemble with encapsulin in vitro [12], suggesting the potential of class P DyPs to act as cargo proteins of encapsulin. A recent high-resolution cryogenic electron microscopy study revealed that a DyP-type peroxidase from *Mycobacterium smegmatis* is a primary cargo protein of mycobacterial encapsulins [89]. Notably, this latter study noted that the encapsulin shell plays a role in stabilizing DyP in a dodecameric form, which is larger than any previously reported DyP oligomer. Considering this packaging of *M. smegmatis* encapsulin as a model system for *M. tuberculosis* would suggest a similar role for DyP from *M. smegmatis* in protecting cells against oxidative stress. By extension, if DyPs from pathogens indeed serve to defend against immune assault from the host, VC2145 VcDyP (class P) from *Vibrio cholerae* [67] might be speculated to have a similar role. One more noteworthy characteristic is that encapsulated DyP has a specified target peptide toward encapsulin. If this is the case, screening for the presence of the target peptide is effective to find unknown encapsulated DyP-type peroxidase [90].

## 5. Perspectives

In this review, we have highlighted the prominent themes in the progression of DyP-type peroxidase research over the past decade. The feature of DyP-type peroxidase that has received the greatest emphasis is the diversity of this family, which displays a widespread expansion of three different classes: P, I and V. Tertiary structural analyses have clearly identified both common and distinct features among the three classes. Substrate specificities and catalytic efficiencies have also been shown to vary among the three classes. Therefore, researchers should continue to apply the current structure–function approach to clarify the catalytic mechanism, which remains unclear. In the end, the catalytic mechanism will likely turn out to be more complicated than that of ubiquitous heme peroxidases, reflecting additional functions such as hydrolase or oxidase activity. One issue in particular that will require further careful study as part of efforts to elucidate the multifunctional feature of DyP-type peroxidase is the wet and dry states of compound I.

Surprisingly, the true physiological roles of DyP-type peroxidases remain unclear; thus, establishing these roles is an important area for future research. As noted above, DyP-type peroxidases have been clearly shown to function in various environments, including extracellular, periplasmic as well as intracellular environments, including within encapsulin. These observations suggest that the physiological role of DyP-type peroxidases is not limited to removal of H_2_O_2_ inside of cells, hinting at other critical physiological functions. In particular, MtDyP and DyP from *B. adusta* seem to have a similar physiological role: defending against counterattacks from the host. Moreover, EfeB and DtpA play important roles in oxidizing Fe^2+^ and Cu^+^, respectively, taking part in the life cycle of their organisms. Compared with other peroxidases reported to date, DyP-type peroxidase have unique features that put them in a class of their own, arguably one that is more advanced than that of ubiquitous peroxidases. With future studies, the universe of DyP-type peroxidase will be unveiled, revealing itself to be widespread and deep like the cosmos.

## Figures and Tables

**Table 1:** Characteristics of representative DyP-type peroxidase from classes P, I, and V.

Class	Former Class	Name	Length (aa)	Reaction with Lignin ^a^	Peroxidase Kinetic Parameters for Anthraquinone Compound				Comp II ^c^	Deduced Radical Sites	Remarkable Comments	Reference
					*K*_m_ (µM)	*k*_cat_ (s^−1^)	*k*_cat_/*K*_m_ (s^−1^M^−1^)	Substrate ^b^				
P	B	YfeX	299									[14]
		DyPPa	299		107	0.024	2.2 × 10^2^	rb5				[15]
		PpDyP	287		40	8.0	2.0 × 10^5^	rb5			manganese oxidizing activity	[16]
		DyP1B	295	✓	120	1.0	9.0 × 10^3^	rb4			manganese oxidizing activity	[17]
		DyP2B	324	✓	Not Detected			rb4			manganese oxidizing activity	[17]
		PfDyP B2	316	✓	10	1.5	1.5 × 10^5^	rb4				[18]
		VcDyP	302		50	1.3	2.6 × 10^4^	rb19		✓		[19]
		*El*DyP	299								manganese oxidizing activity	[20]
		DyPB	350	✓	350	0.05	1.4 × 10^2^	rb4		✓	manganese oxidizing activity (improved in N246A),	[12,21,22,23,24]
											encapsulin carrier	
		DtpB	316									[25]
		Mt-DyP	335								encapsulin carrier	[13,26]
		*Kp*DyP	299						✓			[27]
		TyrA	311		84	5.9	7.0 × 10^4^	rb5				[28]
I	A	EfeB (YcdB)	423								Tat signal, EfeUOB operon, deferrochelatase?	[6,29]
		DyPA	436	✓	210	1.9	9.0 × 10^3^	rb4				[17]
		*Tc*DyP	403		5	41	7.8 × 10^6^	rb19	✓	✓		[30,31]
		*Tfu*DyP	430		29	10	3.5 × 10^5^	rb19			Tat signal	[5]
				✓	179	1.9	1.0 × 10^4^	rb4				[32]
		SviDyP	404	✓				rb19				[33]
		DyPA	428		1,000	13	1.3 × 10^4^	rb4	✓			[21,24]
		DtpA	445						✓		Tat signal, sco, ecuc, Cu-transporter	[7]
		YwbN	416								Tat signal	[8,9]
		FepB	409								FepABC operon (A,EfeO; B,EfeB; C,EfeU),	[10]
											deferrochelatase?	
		*Cbo*DyP	387		17	0.22	1.3 × 10^4^	rb19				[34]
V	C	DyP2	473	✓	48	34	7.1 × 10^5^	rb5			Mn binding site in crystal structure,	[35]
											Mn-dependent oxidase activity	
		AnaPX	469		3.6	384	1.2 × 10^7^	rb5				[36,37]
		SaDyP2	456		61	0.78	1.2 × 10^4^	ab324				[38]
	D	MsP1 (*Msc*DyP)	513	✓							β-carotene is a substrate	[39,40]
		MsP2	510								β-carotene is substrate	[39]
		TAP	504									[41]
		DyP	498		80	980	1.2 × 10^7^				The first found DyP-type peroxidase	[1,2,3,4,42,43,44,45,46,47,48]
		AjPI (*Aau*DyP1)	509	✓	23	114	5.0 × 10^6^	rb5		✓		[40,49,50,51,52]
												[53,54]
		*Egl*DyP	501	✓								[40]
		*Mep*DyP	526	✓								[40]
		*Pleos*-DyP1	516		45	5	1.1 × 10^5^	rb19			manganese oxidizing activity	[55]
		*Pleos*-DyP4	504		82	152	1.9 × 10^6^	rb19		✓	manganese oxidizing activity/	[55]
											Mn binding site is the same as DMP binding site from [43].	
		PsaDyP	516		24	18	7.5 × 10^5^	rb5			oxidase activity/	[56]
											β-carotene, annatto are also substrates	
		PsaPOX	504		Not Detected			rb19			manganese oxidizing activity/alkene cleavage/	[57]
											β-carotene, annatto are also substrates	
		*Il*-DyP4	502	✓	133	5,345	4.0 × 10^7^	rb19			manganese oxidizing activity	[58]
		FtrDyP	484		187	2.6	1.4 × 10^4^				manganese oxidizing activity	[59]
		AncDyPD-b1	511		42	22	5.3 × 10^5^	rb19			ancestral fungal dye-decolorizing peroxidase	[60]
		AjPII (*Aau*DyP2)	unknown	✓	15	256	1.7 × 10^7^	rb5				[40,50]

^a^ include R-478 and model compounds of lignin ^b^ rb and ab mean reactive blue and acid blue, respectively. ^c^ comp II means compound II.
